# A climate for contemporary evolution

**DOI:** 10.1186/1741-7007-8-136

**Published:** 2010-11-11

**Authors:** David Skelly

**Affiliations:** 1 School of Forestry and Environmental Studies, Yale University, 195 Prospect Street, New Haven, CT 06511, USA

## Abstract

A new study of divergence in freshwater fish provides strong evidence of rapid, temperature-mediated adaptation. This study is particularly important in the ongoing debate over the extent and significance of evolutionary response to climate change because divergence has occurred in relatively few generations in spite of ongoing gene flow and in the aftermath of a significant genetic bottleneck, factors that have previously been considered obstacles to evolution. Climate change may thus be more likely to foster contemporary evolutionary responses than has been anticipated, and I argue here for the importance of investigating their possible occurrence.

See Research article: http://www.biomedcentral.com/1471-2148/10/350/abstract

## 

Google 'climate change adaptation' and you will find dozens of websites with an urgent focus on the means by which human societies are considering effective responses to impending changes in climate. Amid this enormous mass of information you will have to work hard to find evidence of the comparatively few researchers considering biological evolution in the same context [1]. Two decades ago, as scientists began in earnest to consider biological responses to changing climate, the dominant theme that emerged focused on the needs for species to either track the climate they favored as those conditions shifted across the landscape, or to tolerate the new conditions *in situ *via phenotypically plastic traits. Today, there is abundant evidence for both modes of response. The movement of species poleward and upslope has been documented; in one review, species were moving an average of 6.1 km poleward per decade [2]. Similarly, evidence for plastic responses abounds [3]. Traits related to the seasonal timing of life-history events (phenology) have undergone widespread change. Such responses may well become the norm. In one study, more than 60% of species considered had undergone measurable changes in either distribution or phenology [2].

Even if the current state of knowledge strongly supports both climate tracking and plastic responses to climate change, why should evolution receive so little attention? The modes of response are not mutually exclusive. There seem to be two main reasons for the lack of interest. First, it is generally difficult to document evolutionary responses. Any observed change in a trait could be evidence of plasticity - and is often assumed to be the result of plastic response - until additional experimental evidence can resolve a non-plastic component. Unfortunately, the absence of evidence has been conflated with evidence of absence in the minds of some researchers. But the second cause may better explain why so few researchers are even looking to evaluate evolutionary responses. In principle, there are some excellent reasons, discussed below, why we might not expect to see many species evolving in response to changing climate.

Bearing on this latter problem is the study published in *BMC Biology *by Kavanagh *et al. *[4], who find strong evidence for an evolutionary response by grayling to differences in water temperatures in streams in Norway colonized by these fish in relatively recent times. The same apparent constraints on evolution are present in such a situation, which makes Kavanagh *et al.*'s evidence for evolution, even though not related to climate change, particularly interesting and important.

## The case against evolution

The most significant knock against evolutionary responses to contemporary climate change revolves around evolutionary rate. Evolution is presumed to be slow because it must operate across multiple generations of species that often have long generation times. Simply put, the environment may be changing too fast for species to respond effectively through evolutionary means. There is some evidence that, particularly within the context of anthropogenic changes, species tend to respond to a shifting environment via plasticity [5]. While it is presumed that the relative rates of environmental change and generation time will jointly influence the capacity of a species to respond, our understanding is still rudimentary.

In addition to being dependent on generational turnover, evolutionary adaptation can be stymied by gene flow. This issue has received enough attention that models aimed at parsing the influences of selection and gene flow on trait evolution constitute their own cottage industry [6] -and for good reason. The ability to resolve the influences of movement and selection is of critical importance to integrating ecology and evolution. Climate change is just one context for this issue. In that case, the individuals in different parts of a species range may be experiencing different climate conditions yet may be connected together by gene flow. Is that gene flow large enough to forestall adaptation to new conditions at the edges of the range? There are few data, but in their absence some researchers presume that gene flow constitutes an additional obstacle to adaptation.

Even if generation time and gene flow were not significant challenges to rapid evolutionary response, there is the problem of available genetic variation. Populations under threat from environmental change are often small. Will there be sufficient genetically based variation in traits to provide the grist for adaptation? There is, in fact, an example from the fruit fly world for which the answer was no. A rainforest fruit fly was found to have no heritable variation for a trait related to desiccation resistance, which, in some situations, has been documented to be under selection [7]. But for the most part, we have no idea. But it is certainly the case that the species at greatest risk of extinction from changing climate are likely to be those with small populations. And it is these species that are likely to have relatively low genetic variation.

## A fish in a perfect storm

Together, generation time, gene flow and a lack of genetic variation constitute a triumvirate of outstanding reasons why climate-change scientists have felt safe in ignoring evolution even as they make sweeping claims about the impending consequences of changing climate on biodiversity [8]. Into this headwind, Kavanagh *et al. *[4] have provided an example of contemporary divergence in a suite of traits that have responded to contrasting thermal environments. Instead of changing climate, these researchers studied the response to thermal variation that emerged when a small number of grayling (*Thymallus thymallus*) colonized the upper reaches of a watershed within which they began breeding in both cold (north facing) and warm (south facing) drainages. Specifically, the researchers studied within a common garden setting embryos and larvae collected from representative warm and cold drainages (two of each). Within this common setting, individuals collected from cold drainages exhibited higher growth rates and higher efficiencies of conversion between yolk mass and hatchling mass. At the same time, cold-drainage animals also developed muscle mass at a higher rate. Collectively, these patterns are consistent with countergradient variation in which selection can promote traits that compensate for an environmental gradient such that the degree of phenotypic variation across the gradient is less than would be expected otherwise [9].

Without additional contextual information these findings might have been deemed interesting primarily as a confirmation of previous results seen in other taxa. The context that yielded divergence, however, makes all the difference in this case. Even as they bred in these contrasting environments, the grayling mingled in a reservoir into which the streams emptied. The timing of the colonization is known (just 22 generations ago), the thermal environments of the drainages are well characterized, and the existence of ongoing gene flow among them has been documented. In spite of a reasonably long generation time (around five years), a small founding population, and ongoing genetic exchange, a suite of traits have diverged in remarkable fashion. In fact, Kavanagh *et al. *have shown that contemporary divergence is possible in spite of the existence of multiple hurdles that are commonly believed to provide good reasons not to consider as significant evolutionary responses to changing climate.

What lessons should climate change researchers take away from the findings of Kavanagh *et al.*? There are two worth highlighting. First, textbook-style descriptions of the possible constraints on adaptation should not be viewed as a good reason not to interrogate systems about the potential for evolutionary response [10]. Arguably, grayling in this study had three strikes against them and yet they showed substantial divergence in critical traits over contemporary timescales. The second lesson is more sobering. None of the embryos in Kavanagh *et al.*'s study were able to tolerate a rearing temperature of just 12°C, even though grayling from other parts of Europe are able to survive at such temperatures. As Scandinavia warms, it is not clear whether the grayling in lake Lesjaskogsvatnet will be able to endure, or whether their relatives from warmer climes will have to supplant them for the species to persist locally.

## Acknowledgements

I thank the National Science Foundation, the David P Garmany Fund of the Hartford Foundation for Public Giving, and the Army Research Office for support of my research.
